# Pore structure evolution in andesite rocks induced by freeze–thaw cycles examined by non-destructive methods

**DOI:** 10.1038/s41598-022-12437-5

**Published:** 2022-05-19

**Authors:** M. Maľa, V. Greif, M. Ondrášik

**Affiliations:** 1 Department of Engineering Geology, Hydrogeology and Applied Environmental Geophysics, Faculty of Natural Sciences, Comenius University, Bratislava, Slovakia; 2 Department of Geotechnics, Faculty of Civil Engineering, Slovak University of Technology, Bratislava, Slovakia

**Keywords:** Geology, Mechanical properties

## Abstract

In this paper, we compare the values of petrophysical properties before and after 100 freeze–thaw (F–T) cycles, as well as recorded length change behaviour and temperature development on a vacuum-saturated fractured andesite rock sample taken from the Babina Quarry in Slovakia using a specially-constructed thermodilatometer, VLAP 04, equipped with two HIRT-LVDT sensors. We also used non-destructive visualization of the rock pore network by µCT imaging in order to study the development of the pore structure and fracture network in pyroxene andesites during the freeze–thaw process. The results show that the andesite rock samples, due to good fabric cohesion, low porosity, and low pore interconnection, showed good resistance against frost-induced damage. However, it must be stated that the main process causing disintegration of this type of rock is fracture opening, which is caused by internal stresses induced by water–ice phase transition. The overall residual strain recorded after 100 F–T cycles was not significant, however, the increase of 31 pp in volume of the fracture showed us that repeated freezing and thawing can lead to long term deterioration in terms of subcritical crack growth in brittle-elastic solids like pyroxene-andesite rocks.

## Introduction

Mechanical or physical weathering (physical rock breakdown), or more specifically, frost weathering induced by repeated freeze–thaw cycles, is a key process that influences the appearance of geo-relief, especially at high altitudes or in polar periglacial regions. It is closely related to the regional and global climate^1^. Physical rock breakdown mainly stems from the propagation of cracks in the rock matrix^2^. The sources of stresses which are induced by freezing and thawing (further only F–T cycling) and cause deterioration of rock material are still discussed^3^. Frost damage may include a combination of several mechanisms and is currently attributed mainly to crystallization pressure^4–10^, subsequent hydraulic pressure^10^, and volumetric expansion^11^. One or another usually predominates depending on material properties, moisture conditions, and thermal conditions^12^.

Ice crystals tend to grow in thermodynamical disequilibria^3, 6, 13^. This crystal growth exerts crystallization pressure on the pore wall through a nanometer-thick liquid layer, which is present between two solid phases (ice crystal and pore wall). In a cylindrical pore (it is not generally applicable), the crystallization pressure—Pc is represented by the tensile hoop stress written in Eq. (1)^6^ as follows:

1 $$P_{C } = \frac{{\gamma_{CL} }}{{\left( {r_{p} - {\updelta }} \right)}}$$

where P_A_ is additional pressure provided by the pore wall; γ_CL_ is the crystal/liquid interfacial free energy; r_p_ is the radius of the cylindrical pore and δ is the thickness of the water film between the ice crystal and the pore wall (≈ 0.9 nm).

According to the *theory of hydraulic pressure*^10^, water crystallizes first in the larger pores of the system. There are two possible development scenarios. The first one is that the 9% volume expansion^11^ causes part of the water in these larger pores to be forced out to the smaller pores in the vicinity. Therefore, if this expelled water cannot find a pore large enough to relieve pressure, hydraulic pressure builds up. The process is called cryosuction and was first quantified by Everett^4^. Cryosuction could be explained by a shortened version of Clausius–Clapeyron Eq. (2) as follows:

2 $$P_{c} - P_{l} = \frac{{\rho_{l} L_{f} }}{{T_{m} }} \left( {T_{m} - T} \right)$$

where P_c_ and P_l_ are the ice and water pressure, ρ_l_ is the density of the water, L_f_ the specific latent heat of fusion of the ice at the bulk freezing temperature T_m_ and the atmospheric pressure and T the total temperature of the system.

During this process, the ice front is progressing until it encounters a temperature under which it cannot further penetrate the surrounding pores of the network. The water which remains in supercooled state in smaller pores serves as a water reservoir for further crystallization. In the second scenario, unfrozen water migrates towards ice crystals in macropores driven by the difference of chemical potential between ice and water.

Based on poromechanics models^3^ the total stress is related to the crystallization pressure P_c_ according to Eq. (3). In a case that it exceeds the local tensile strength of the frozen rock, a cracking of the rock material will occur (4).

3 $${\upsigma } \approx {\text{b*}}S_{C} *P_{C}$$

4 $${\upsigma } > \frac{{{\upsigma }_{z} }}{{\sqrt {3\left( {1 - 2{\upnu }} \right)} }}$$

in which b is the Biot coefficient; S_c_ is the ice crystal saturation degree; σ_z_ the tensile strength and ν the Poisson's ratio. Ruedrich and Siegesmund^14^ emphasized the importance of water saturation in the frost weathering process. The saturation in turn depends on various material properties, such as mineralogical composition^15^ and petrophysical properties—the porosity, pore size distribution, or permeability^16–18^. The higher the saturation degree, the higher is the risk of stress and subsequent damage induced by the crystallization pressure, due to the larger volume fraction of ice in the rock pore system. Other important material parameters that co-define F–T resistance are mineral composition, tensile strength, and anisotropy^19^. Materials in which moisture is below the critical degree of saturation also tend to degrade over time due to exposure to repeated F–T cycles. However, the weakening of the material is induced by stresses lower than the strength of the material. This phenomenon is referred to as subcritical crack growth^20–23^ or fatigue cracks^12, 24^.

As it is clear from the above-mentioned studies, sufficient information about the mass characteristics of the pore environment is one of the key components of understanding the mechanism of frost damage in rock or any porous material. The main goal of this paper is to quantify the petrophysical parameters of the pore network system of the tested andesite before and after F–T cycling. The hypothesis is based on the assumption that damage mechanisms induced by ice crystallization in vacuum—saturated fractured andesite specimen will result in significant microstructural changes of pore network parameters—specifically increase in porosity, changes in pore size distribution, increase in pore interconnection and subsequent residual strain.

Methods of frost damage assessment in rocks are based on parameters obtained mainly by destructive testing of samples. On the other hand, we used modern, non-destructive experimental laboratory and visualization techniques, such as the spontaneous imbibition method^25, 26^, a newly-developed indicative rock pore structure identification method^27^, and X-ray µTomography based on non-destructive visualization. These methods were applied in this combination applied for the first time in a study of weathering. The study focused on the change in andesite rock pore distribution triggered by F–T cycling, and special attention was placed on a single transverse crack present in the tested specimen. The novelty of the proposed paper is found in the integration and comparison of strain measurement and temperature monitoring carried out by the specially constructed thermodilatometer VLAP 04 with the above-mentioned nondestructive techniques. Using VLAP 04 we were able to identify the production and diffusion of latent heat manifested by the temperature spike and determine its intensity and duration, as well as determine the strain pattern by LVDT sensors. Understanding fracture dynamics in porous media in relation to pore space is essential in environmental and material studies of hard rocks in natural conditions, as well as rocks used as building material, since fractures can compromise the structural integrity of the exposed materials^28^.

One of the new tools for quantification of the pore space evolution of the specimen caused by freeze–thaw action is also X-ray computed microtomography (µCT)^28–30^. The main advantage of this method is the non-destructive character, 3D visualization, localization, and evaluation of internal structural changes prior to and after F–T cycling. Dewanckele et al.^31^ employed µCT for studying the effect of frost damage on limestones at the microscale. According to his study, freezing and thawing depends significantly on the presence of microfacies in limestone, and thus on the existence of pre-existing flaws. The results also showed that the pore size distribution affects the location of crystallization spots inside the tested rock. In a separate study, Park et al.^32^ used µCT for studying igneous rock samples. They scanned them before and after subjecting them to a laboratory-controlled freeze–thaw test. According to their study, frost susceptibility mainly depends on porosity and tensile strength of the tested rocks. De Kock et al.^28^ worked with state-of-the-art µCT as well. They studied the fracture propagation and pore-scale dynamics as a response to repeated freezing and thawing. In their work, they observed the progressive discontinuity opening during the freezing period, while during the thawing period, it closed again. After a certain number of cycles, it stopped closing during melting, and did not expand further during freezing. According to their hypothesis, this demonstrated the effect of a stable degree of saturation during the freeze–thaw experiment, because after a certain number of F–T cycles, sufficient space was created for the ice to grow indefinitely. After adding water to the system, crack growth continued due to frost wedging.

Continuous F–T cycling is capable of progressively generating more microcracks in the matrix of the rock material. Once a critical threshold is exceeded, microcrack growth will progress rapidly over a small number of F–T cycles, and this causes nonlinear material decay^33^. The rate of crack growth depends mainly on the mechanical and petrophysical properties of the material.

## Material and methods

### Initial rock fabric properties

**Figure 1:**
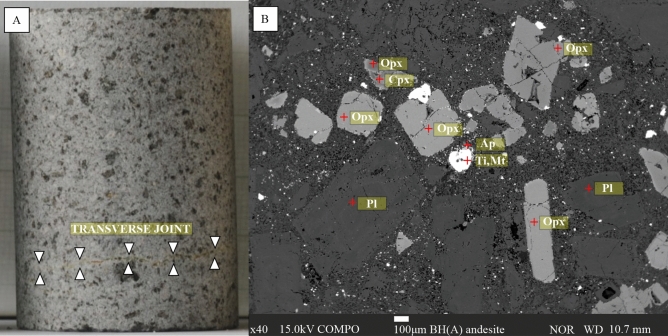
(**A**) Macro photo of BH(A) specimen with transverse joint; (**B**) Mineralogical composition on standard thin section by SEM.

The propagation of microcracks is a significant phenomenon of the weakening of brittle crystalline rocks with usually high mechanical strength; this is the main reason why we decided to work with the rock core of pyroxene rich andesite (Fig. 1a), having a size of 51 mm ± 1 mm in length and 32 mm ± 1 mm in diameter, which was cut and machined from a specimen block collected from the Babina–Hanišberg quarry—BH(A) type.

The rock fabric is one of the main factors which controls petrophysical properties and material behaviour during the weathering process. Mineral content analyses were performed by SEM—Scanning Electron Microscopy of standard thin sections. Andesite from the Babina quarry Hanisberg is coarse, amphibole-pyroxene porphyritic andesite. The main components are orthopyroxene, clinopyroxene, and plagioclases with basic accessory minerals like apatite or titanite (Fig. 1b).

Igneous rocks, which formed by cooling and subsequent crystallization from magma or lava, undergo considerable contraction during the cooling period. This gives rise to tensile forces strong enough to break the rock into several jointed blocks. Such contraction or shrinkage is generally accepted to be the cause of the vertical type of joints in granites, as well as the well-known columnar joints in andesites and other effusive rocks. These oriented joints often serve as preferential weakness planes in various mechanical weathering processes. Therefore, in our research, we focused on a sample with a visible transverse joint, parallel to the bedding of the studied andesite (Fig. 1a).

**Figure 2:**
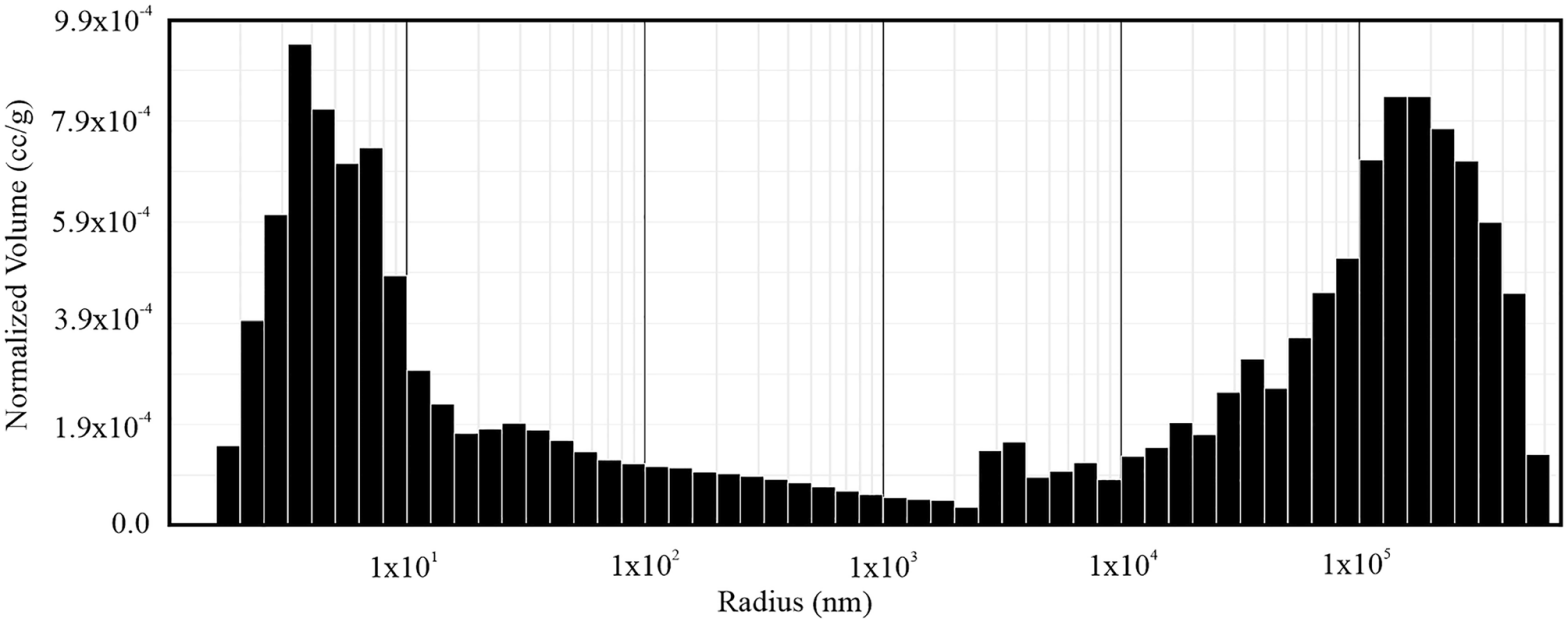
Pore size distribution by mercury intrusion porosimetry showing bimodal pore size distribution pattern.

As was previously mentioned, rock fabric is an important factor that affects and controls the topologic and geometric properties of the pore system. Various researchers have reported that the petrophysical properties are important rock parameters that significantly affect the frost resistance of rocks. To quantify the changes in pore systems induced by repeated freeze–thaw cycles, it is necessary to provide detailed information about total porosity and pore size distribution. It is important not only because of the amount of water that is absorbed in the rock matrix and pore system, but also due to the possible amount of ice that will crystallize in given environmental conditions, and thus, for the interpretation of the internal frost weathering damage mechanisms. In order to provide detailed information about initial parameters, we applied standard destructive procedures^34, 35^ applicable for the determination of bulk density, total porosity, and pore size distribution. Representative pore radii distribution was measured by standard mercury intrusion porosimetry (MIP). Based on data from MIP, we can state that the pore size distribution pattern of these andesites is bimodal, which means that this lithological type is characterized with two maxima: the first one in larger capillary pores and the second in pores with a small radius. The BH (A) andesites are characterized by a large number of pores smaller than 1 × 10^1^ nm, while the second one has a large number of pores larger than 1 × 10^5^ nm (Fig. 2).

Changes in geometrical and topological parameters of pore space after repeated freeze–thaw cycles can be quantified by non-destructive techniques, specifically helium pycnometry (effective porosity), spontaneous imbibition testing (pore interconnectivity), and the experimental rock pore structure identification method (pore size distribution). In essence, these techniques appear very valuable; however, they are not standardized because they are relatively quick to perform, cause no damage to the sample, and can be carried out repeatedly on the same specimens. Therefore, they are suitable for evaluation of the effect of frost on a certain type of porous material.

Effective porosity was analyzed by the pycnometric method, which is described as helium pycnometry. Helium is used in helium pycnometers to determine the particle density (or specific weight) of pulverized samples. Helium pycnometer was applied to determine rocks' effective porosity^36^. The measurement is based on the Archimedes principle, but instead, water is substituted by inert technical high-purity helium replaced by the sample volume Vx in the test chamber. After putting a cylinder-shaped rock sample into the chamber of known volume VC, gas is let in till the required pressure is achieved. When the inlet is closed, gas penetrates all effective pores of the rock or other porous sample until the equilibrium pressure p1 is reached. After opening the vent to another/additional chamber of known volume VA, helium expands and pressure drops until a new equilibrium at the pressure p2 is reached, following the equation:

5 $${\text{p1}} \cdot {\text{V1}} = {\text{p2}} \cdot {\text{V2}}\quad \left( {{\text{Boyle}}^{\prime}{\text{s}}\;{\text{law}}\;{\text{of}}\;{\text{gas}}\;{\text{expansion}}} \right).$$

The volume of a solid object can be calculated from this equation as a function of the ratio p1:p2. The custom evaluation method using a precise calibration curve was developed. For rock sample testing, calculated volume Vx represents the solid phase volume (without helium accessible pores, i.e., He-effective pores). However, closed pores are included in Vx. Afterwards, the volume of the effective pores is calculated as V_efHe_ = V − Vx, where V is the total (or “envelope”) volume. Regularly-shaped cylindrical samples are used, volume V is calculated from their dimensions.

Before each sample measurement, the sample was intensively washed by helium flow. One measurement is interpreted from the mean of 10 steady ratios p1:p2, with maximum deviation ± 0.001.

#### Pore size distribution

For the identification of the rock pore structure, two different methods were used. As was stated above, representative pore radii distribution was measured by standard mercury intrusion porosimetry according to STN 72 1011^35^ in addition to a newly-developed experimental rock pore structure identification method^27^. The new method is based on the assumption that water sorption (adsorption and absorption) into rock pores is controlled by rock pore structure; thus, water sorption under controlled conditions determines the rock pore structure. The controlled conditions are achieved by three different suction tests: 72 h water vapor adsorption at 98% RH, 48 h water absorption under atmospheric pressure, and 24 h vacuum water absorption. In this way, four different rock pores types can be identified according to their size and accessibility to water: (6) micropores and mesopores, (7) easily-accessible macropores, (8) partially accessible macropores, and (9) closed pores of any size. The pore size classification, micropores, mesopores and macropores is the one by IUPAC (International union of Pure and Applied Chemistry)^37^. The main advantage of this method is its nondestructive character and ability to be applied repeatedly prior, during, or after rock testing.

The 72 h water vapor adsorption test at 98% RH was performed to identify the content of micropores and mesopores in the rock samples. Prior to the adsorption test, the sample was dried in an air circulating oven at 105 °C and weighed with the accuracy ± 0.001 g. Dry sample was put into airtight chamber with 98% RH and constant temperature (21 °C). The 98% RH was maintained by using a super-saturated solution of hydrated copper sulphate (CuSO_4_·5H_2_O)^38^. After 72 h, the sample was individually weighed without removing from the climatic chamber. From the rock weight difference, the content of micropores and mesopores was calculated according to Eq. (6). The 48 h water absorption test made according to STN EN 13755^39^ was used to identify macropores that were easily accessible to water. The 24 h water vacuum absorption test was further performed in order to identify macropores that were partially accessible to water. Finally, the content of closed pores was identified from the total rock porosity determined according to STN EN 1936^34^. In order to calculate the content of micropores and mesopores (M_P_), easily-accessible macropores (N_BULK_), partially accessible macropores (N_VOID_), and closed pores of any size (N_C_) from the test results following equations were used:

6 $$M_{P} = \, N_{AD98} \quad \left[ {{\text{wt}}.\% } \right]$$

7 $$N_{BULK} = \, N_{48} {-} \, N_{AD98} \quad \left[ {{\text{wt}}.\% } \right]$$

8 $$N_{VOID} = N_{V} {-} \, N_{48} \quad \left[ {{\text{wt}}.\% } \right]$$

9 $$N_{C} = n*\frac{{\rho_{w} }}{{\rho_{d} }} - N_{V} ,\quad \left[ {{\text{wt}}.\% } \right]$$

where N_AD98_ is the content of adsorbed water, N_48_ is the content of absorbed water after 48 h saturation under atmospheric pressure, N_V_ is the content of water saturated into pores vacuum, n is the total porosity in vol.%, ρ_w_ is the density of water, and ρ_d_ is the apparent density of dry rock.

### Pore connectivity by the spontaneous imbibition method

**Figure 3:**
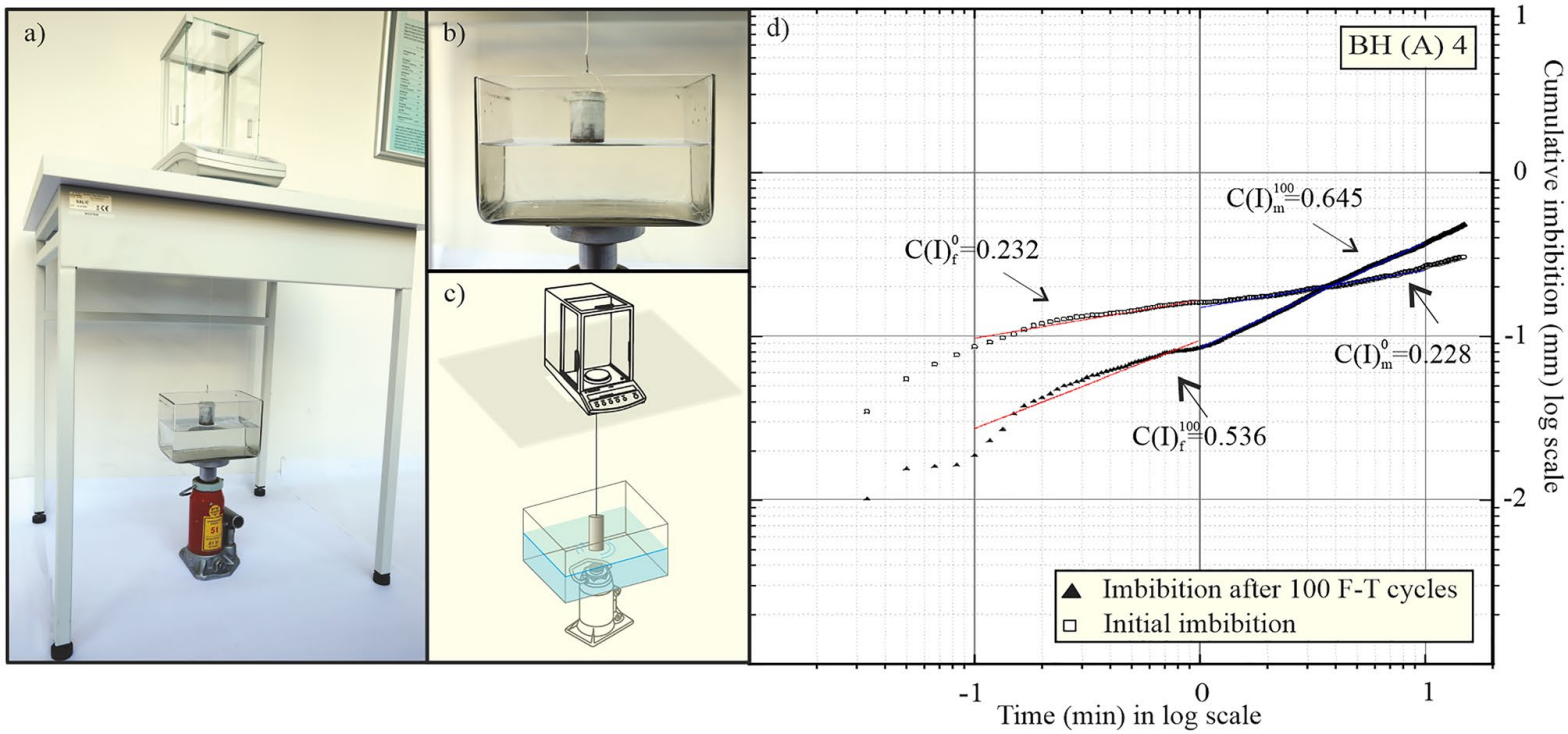
Overall view of the equipment used for spontaneous imbibition tests (**a**) with details of the water tank placed on the jack (**b**) and schematic drawing of the test arrangement (**c**); typical imbibition curve for BH(A) 4 specimen showing division for fast (0.1–1 min) (red) and medium (1–10 min) (blue) time intervals.

The method of spontaneous imbibition is a simple, nondestructive test procedure for determination of the connectivity of rock pores, which is an important topological parameter of pore systems. During the test, one face of the sample is exposed to water, where the mass of water uptake is measured over time^25^. It exploits the analogy with diffusion, where, in homogeneous materials (without taking gravitational effects into account), the distance to the wetted front increases with the square root of time l ~ t^0.5^. In this work, a modified testing procedure to the one employed by Hu et al.^25^ was adopted to mitigate for any errors due to buoyancy force or effect of evaporation in such a way that the specimen was tested in a larger water tank compared to a Petri dish by Hu et al.^25^. The specimen was dried at 105 °C for 48 h and then wrapped in PE foil, except for the base of the core sample, including a 1 mm side wall exposed to water in order to avoid evaporation losses. The sample was then stored in a desiccator and tested suspended under a balance with automatic reading and recording of data every 1 s for the duration of 15 min. A glass water reservoir with dimensions of 202 × 122 × 125 mm filled with distilled water up to 65 mm height was placed on a supporting jack to bring the rock specimen in contact with the water in the reservoir, and the sample was submerged to a depth of about 1 mm (Fig. 3a–c). The temperature was maintained at 23.0 ± 1 °C. Imbibition tests (SI) were carried out in triplicate on the sample. The sample was dried at 105 °C for 48 h between tests. Each test was analyzed by plotting cumulative imbibition height (mm) against square root of time (min) in a log scale. The apparent slope of the linear regression curve C(I) provides an imbibition slope, thereby characterizing the speed of water imbibition into the sample. This relation, which is originally based on Handy’s^40^ model, is valid only for small size samples where the effects of gravity are minimal—this is also the case here. Two distinct imbibition slopes were determined separately for the time intervals 0.1–1 min and 1–10 min. Imbibition slopes for fast 0.1–1 min interval were marked $C\left( I \right)_{f}^{0}$ for initial state and $C\left( I \right)_{f}^{100}$ after 100 freeze–thaw cycles. Slopes in the medium time range 1–10 min were marked $C\left( I \right)_{m}^{0}$ and $C\left( I \right)_{m}^{100}$ respectively (Fig. 3d).

### Non-destructive visualizations

Non-destructive visualizations of pore space and fracture propagation was performed on High-energy micro-CT Phoenix v | tome | × L 240. The acquired images are based on the attenuation of the X-ray through the investigated specimen. The design of industrial µCT, unlike clinical, is based on a fixed X-ray tube and a fixed detector, between which the sample rotates, and higher radiation energy > 12.4 keV is used. In our work, we used a voltage of 170 kV. With the rock cores of size 51 mm ± 1 mm in length and 32 mm ± 1 mm in diameter, the voxel size was equal to 34 µm, so we were able to determine only macropores within the sample. The sample had to be scanned 2 times—before and after 100 F–T cycles; the main goal of this procedure was to match the 3D structural information of each scan. For one sample, a total of 2200 projections were registered. To reduce the noise factor, 1 frame was taken from each projection with an exposure time of 500 ms. The data were reconstructed by using the phoenix datex 2-reconstruction program and then exported to a vgl format. VGS studio MAX 2.2 software was used for the final visualization of the pore space of the specimen with a transverse joint before and after F–T testing.

The acquired images were processed using several 3D visualization techniques. Quantitative data, such as porosity and pore size distribution, were derived using these methods and compared with the above-mentioned methods (e.g., spontaneous imbibition method, helium pycnometry). In the first step, it was necessary to distinguish the pore space from the matrix and mineral grains by binarizing the obtained images. For this purpose, the method of thresholding was used. The selected thresholding method was based on visual feedback. Binarization transforms a gray-level image into a binary image. This method is used when the relevant information in the gray level image corresponds to a specific gray level interval. In the output binary image, all pixels with an initial gray level value that were lying between the two bounds were set to 1, all the other pixels were set to 0. In addition, because the pore separation method is prone to errors in the presence of noise, we applied despeckle filtering to remove noises generated by the thresholding method. Image binarization was firstly performed on 2D ortho-images and then used to generate a 3D image, which was further processed. The interconnected pore space was separated from the binary images using the concept of the watershed segmentation algorithm. The principle of the watershed algorithm is to compute watershed splitting lines on a segmented 3-D image, which would detect surfaces and separate agglomerated particles, which are subtracted from the initial image^28^. The result of the analysis was then determination of the porosity and the pore size distribution of pore systems. The results were processed in the form of logarithmic histograms and visualizations.

### Freeze–thaw test

After completion of spontaneous imbibition testing, the specimen was unwrapped, dried at 105 °C for 24 h, its weight was measured, and thereafter vacuum-saturated with distilled water for 48 h to obtain maximal water saturation. The sample was further weighed, wrapped in PE foil to prevent evaporation, and placed inside the thermal chamber of the thermodilatometer VLAP04. The VLAP04, which is used for the frost damage test, is able to control the temperature in the range from − 17 to + 60 °C. A control thermocouple from the thermal chamber was placed along the central rotational axis of the cylindrical dummy sample, which was made from the same material as the tested specimen, and the temperature was cycled from − 10 to + 10 °C. The specimen was subjected to 100 freeze–thaw cycles with a cooling rate of − 0.18 °C/min and heating rate of 0.21 °C/min. The length change of the specimen was measured using LVDT (linear variable differential transformer) sensor HIRT-LVDT-T101 F with an accuracy of ± 1 × 10^–3^ mm. The temperature inside the sample and strain was constantly monitored and recorded in 1 min intervals, which allowed for monitoring of dilatometric behaviour of the specimen during the phase transition of water in the pores.

## Results

### Petrophysical properties

#### Porosity

The mineralogical composition and fabric (structure and texture) determine the rock’s petrophysical properties. The structure of the pore system of andesite from Babiná is typical for extrusive crystalline igneous rocks. Their primary porosity is very low, and they are characterized by secondary porosity in the form of cracks, the extent of which depends on the time and extent of the temperature processes associated with the cooling of the lava.

The total amount of water adsorbed into the pore system is closely related to the porosity of the rock and was calculated as the degree of saturation Sr (%) before and after 100 F–T cycles with respect to the total water absorption under vacuum of the respective sample according to the following equation:

10 $${\text{Sr}} = \left( {\frac{{{\text{N}}*{{\rho }}_{{\text{b}}} }}{{\text{n}}}} \right) * 100$$

where N is the sorptivity; ρ_b_ is the bulk density and n is the total porosity of the sample. Sr before cycling was 69.4% and increased to 98.02% after 100 F–T cycles. It might seem that after vacuum saturation, the whole volume of pores represented by effective porosity is filled with water, which is not the case. During vacuum saturation, only pores which are big enough for a molecule of water to enter the pores and are somehow interconnected to the surface of the sample are saturated. During F–T cycling, the pore interconnection, increased and that was the reason that we were able to measure a higher degree of water saturation after repeated vacuum saturation, so the difference between Sr determined before and after 100 F–T cycles is the evidence of mechanical stresses within the sample.

Initial values of porosities and the degree of saturation, as well as their changes after F–T cycling can be found in Table 1.

**Table 1:** Total and effective porosity, mean values of imbibition slopes C(I) from triplicate measurements and degree of saturation of BH(A) 4 specimen before and after F–T cycling.

Porosities and degree of saturation of BH(A) 4 specimen			
	Before F–T	After F–T	Δ (%)
n (%)	Apr-15	Apr-27	0.12
n_ef_ (%)	0.57	02-Jul	01-May
Sr (%)	69.4	98.0	28-Jun

Imbibition slope (CI) of BH(A) 4 specimen			
$C\left( I \right)_{f}^{0}$	$C\left( I \right)_{f}^{100}$	$C\left( I \right)_{m}^{0}$	$C\left( I \right)_{m}^{100}$
0.184	0.471	0.194	0.708
$\Delta {\text{C}}\left( {\text{I}} \right)^{f}$ (%)		$\Delta {\text{C}}\left( {\text{I}} \right)^{m}$ (%)	
155.26		264.83	

#### Pore radii distribution by the indicative rock pore structure method

**Figure 4:**
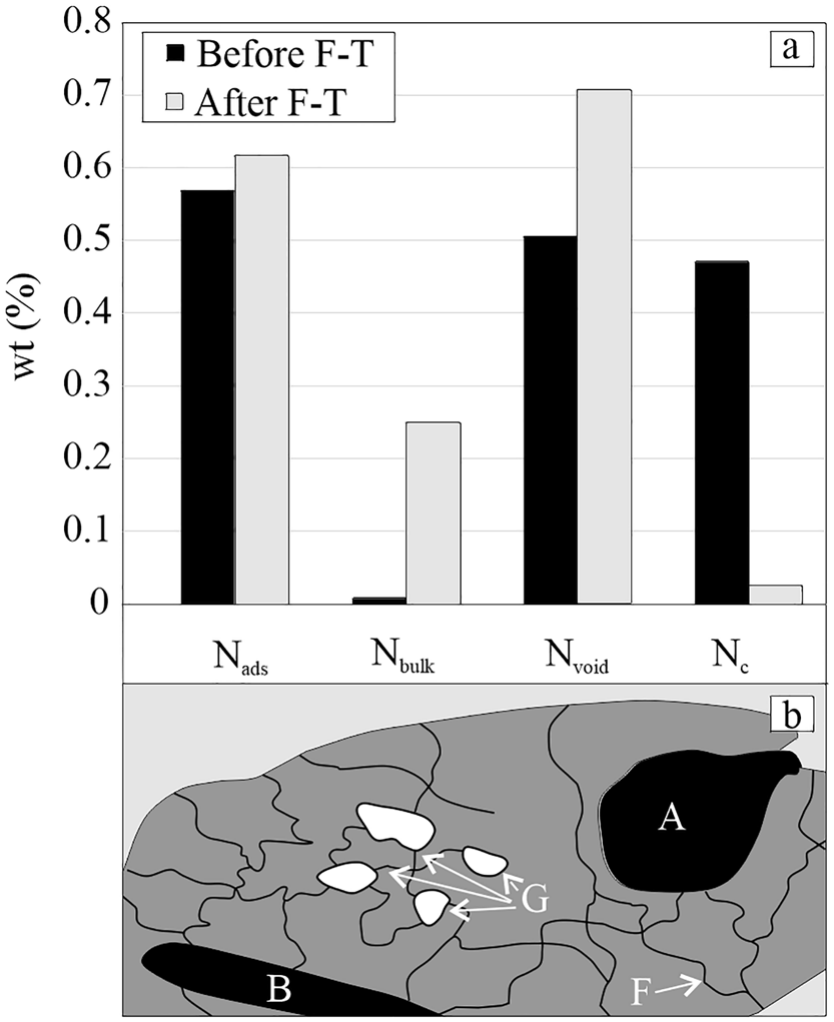
Pore size distribution by indicative rock pore structure method before and after 100 F–T (**a**) Illustrated pore structure of the BH(A) andesite (**b**): (A) N_void_—pores with narrow necks, (B) N_void_—blind pores; (G) N_c_—isolated pores which are not accessible to water; (F) N_ADS_—micropores and mesopores.

The andesite sample from Babina has a specific and complicated rock pore structure (Fig. 4b). Our results show that most of the pore volume consists of pores that are hardly accessible to water (N_void_ = 0.50 wt%), which are represented by macropores of size greater than 50 nm (Fig. 4b-B), and by pores containing adsorbed water (N_ads_ = 0.569) (Fig. 4b-F) (this corresponds to the MIP results). The first pore type is represented by inkbottle-shaped macropores, or large macropores (cavities) surrounded and communicating with the rock surface (the source of water) via micropores, mesopores, or much smaller macropores which create obstacle for water intake into the cavities. Water can enter into these macropores only if the three-phase system is disturbed, because capillary suction does not release water from smaller pores into larger macropores, and the air cannot escape from the inkbottle macropores. The pores which are easily accessible to water are in fact not present in BH(A) 4 andesite (N_bulk_ = 0.008) (Fig. 4b-A). Thus, we can state that the rock pore structure of BH (A) 4 sample is made of a large number of blind pores and isolated pores, which are interconnected by micropores and mesopores. After 100 F–T, the rock pore structure of the test sample changed significantly. A significant increase in the volume of pores which are easily accessible to water is most likely related to crack growth and increase in the hydraulic conductivity of the sample in terms of increasing pore interconnection. The volume of N_ads_ micro and mesopores also increased slightly, as did the volume of pores which were hardly accessible to water. However, the volume of isolated Nc pores decreased significantly by up to 0.446 wt% (Fig. 4a). This difference corresponds to the sum increment of N_bulk_ and N_void_ which is 0.242 + 0.203 = 0.445 wt%. Theoretically, this may suggest that due to F–T cycling, the volume of water-isolated pores changed in equal proportions to the volume of N_bulk_ and N_void_ pores. Such a process is perhaps a consequence of subcritical crack growth by a significant microcrack opening.

#### Pore connectivity

The spontaneous imbibition test carried out on andesitic sample, which had been subjected to 100 freeze–thaw cycles, showed an increase in the imbibition slope, thus indicating an increased rate of water uptake for both fast and medium time intervals. In a fast timeframe, the average value of $C\left( I \right)_{f}^{0}$ = 0.184 increased by 155.26 pp. to an eventual 0.471 after application of F–T cycling. For the medium time scale, the imbibition slope increased even more by 264.8 pp. from initial $C\left( I \right)_{m}^{0}$ = 0.194 to a final value of $C\left( I \right)_{m}^{100}$ = 0.708 (Table 1). A considerable standard error resulting from the F–T cycling in andesite needs to be noted, since it is an igneous rock with extremely low porosity. F–T cycling generated either new, or widened already existing cracks, forming preferential paths for water uptake; therefore, a higher apparent imbibition slope in the tested andesite sample was observed.

#### Freeze–thaw tests

**Figure 5:**
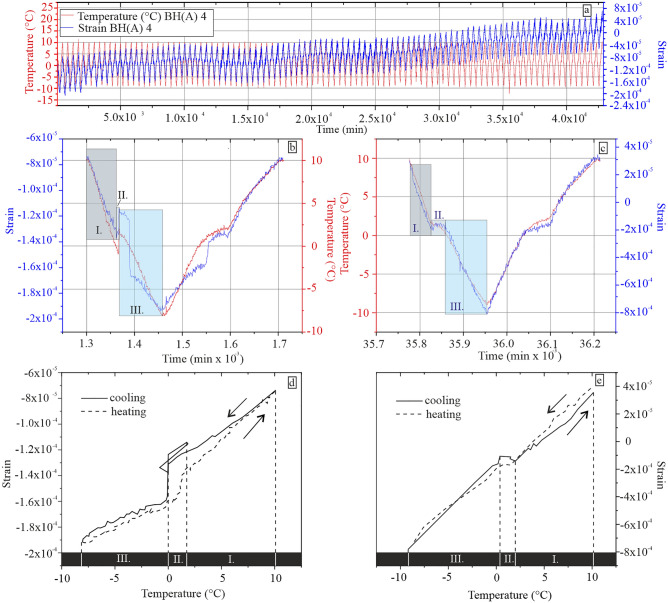
Record of temperature and strain behaviour of BH(A) sample; (**a**) Temperature and strain path during 100 F–T cycles; (**b**,**c**) Detailed records of temperature and strain vs time during 4th and 83rd F–T cycle; (**d**,**e**) Detailed records of strain vs. temperature during 4th and 83rd F–T cycle.

The strain behaviour resulting from ice crystallization of the water-saturated sample is based on various pore system characteristics, such as the water content in rock pores, rock fabric and mineralogical composition. The strain and temperature behaviour during F–T cycling is further plotted as strain versus time diagrams in Fig. 5 and divided into three characteristic zones—I; II; III, by applying a modified division by Ruedrich and Siegesmund^14^. This allowed us to make a conclusion about the effective mechanisms of the deformation. Above the freezing point, the strain curve was in zone I. This process is not yet affected by ice crystallization in the rock pores. The contraction in zone I continues with the expansion of the specimen in zone II. The phenomenon of specimen expansion can be traced back to the initial crystallization of ice in zone II. Since crystallization is an exothermal process, it is associated with the release of energy in the form of latent heat. During the crystallization (nucleation), temperature remains constant, and after its completion, the temperature starts to decrease again. During ice crystallization, there is a 1 nm thick liquid layer present between ice crystal and the pore wall, which causes the ice crystal to exert pressure on the pore wall^6^. The liquid layer sustains itself by disjoining van der Waals forces between the crystal and the pore wall with a magnitude of several tens of MPa^41^. At the same time, due to thermodynamic imbalance, water is attracted to the forming ice front. At 0 °C, the water is supercooled in the micro and mesopores and serves as a water reservoir for the forming ice front. The migration of water to larger pores, where ice crystallizes, causes hydraulic pressures on the pore walls (if the pores are small enough or pore interconnection is low) and at the same time, causes reduction in pore pressures (negative capillary pressures) in smaller pores. This reduction resulted in the shrinking of the material, which explains the strong contraction of specimen in zone III. However, the ice crystals found in the macropores continue to grow, causing further generation of crystallization pressures on the pore walls. On the other hand, crystallization pressures can overcome negative capillary pressures only if the thermodynamic equilibrium is disturbed^13^. After 100 F–T cycles, a residual strain of 8 × 10^–5^ was observed (Fig. 5). Residual strain with a value of 8 × 10^–5^ is probably caused by subcritical crack growth. This process can lead to long-term deterioration of the material, which normally exhibits relatively high mechanical strength.

### Non-destructive visualizations

**Figure 6:**
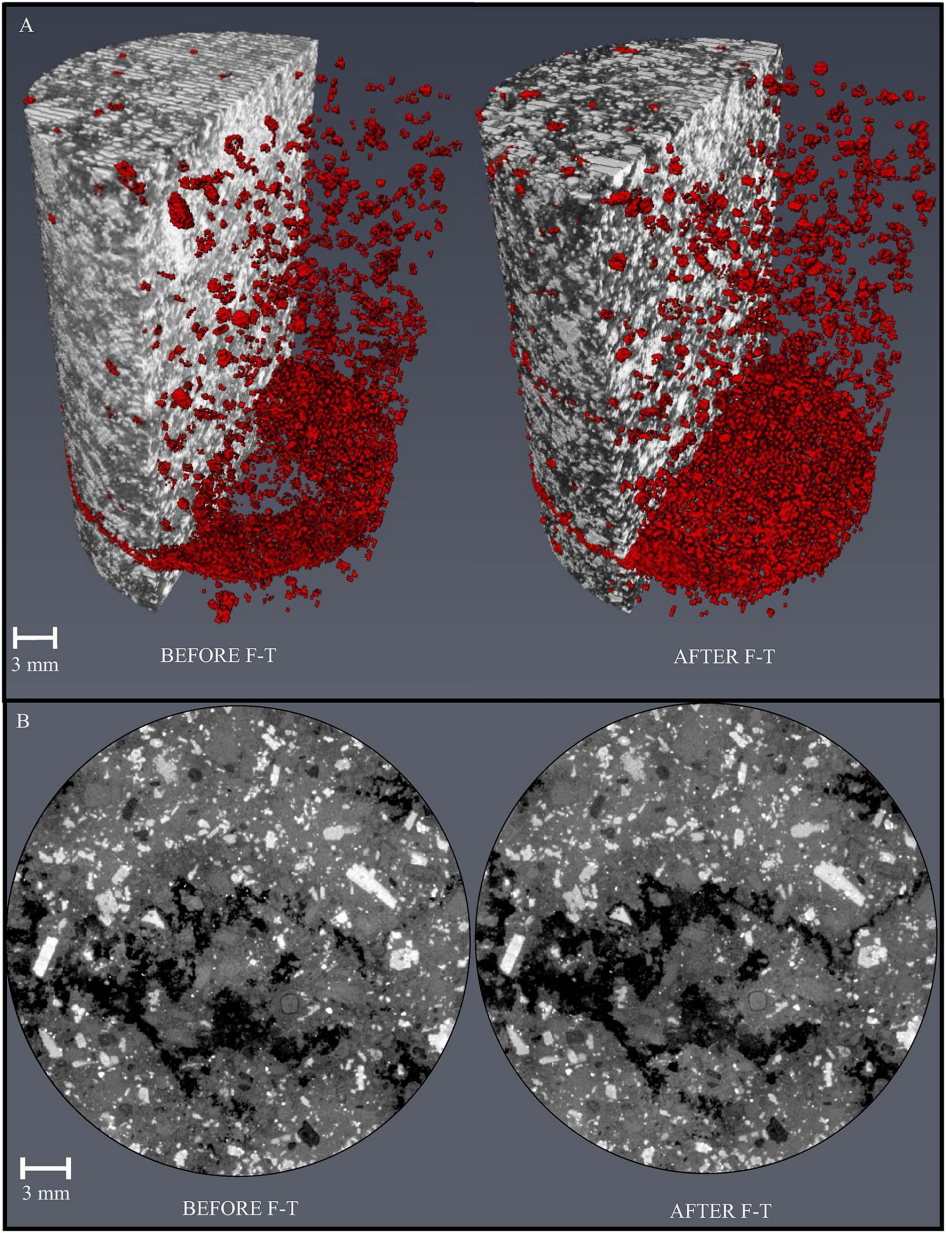
(**A**) Distribution of voids before and after 100 F–T cycles by X-ray µtomography showing changes in porosity as an expression of F–T decay; (**B**) Fracture on 2D orthoslices before and after 100 F–T cycles.

100 F–T cycles with temperature oscillations from 10 to − 10 °C results in the microstructural evolution of pore spaces and leads to the development of fractures. Dimensional changes are obtained by measuring the equivalent diameter (ED) of each pore structure before and after F–T cycling. This parameter represents the diameter of a sphere with the same total volume as measured object. The defect volume distribution as a function of position in X, Y, Z-directions is shown in Fig. 6.

**Figure 7:**
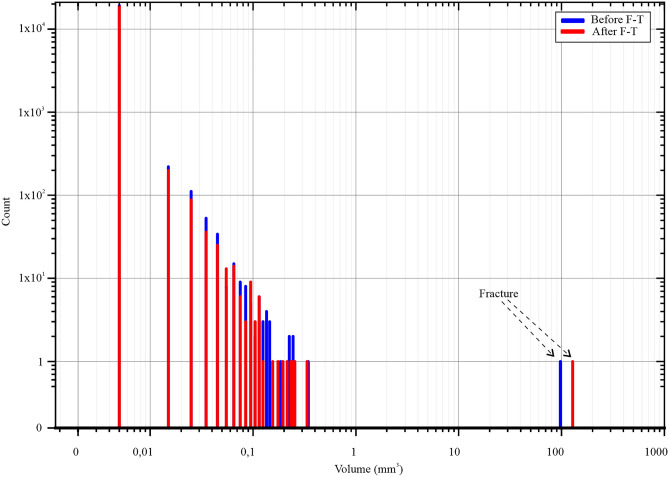
Histogram showing pore size distribution of macropores (due to the used voxel size of 34 µm/voxel, we were able to scan only pores with an effective diameter larger than 136 µm) in andesite determined by Micro X-ray tomography before and after 100 F–T cycles.

Total volume of detected pores was 127.29 mm^3^, which means total porosity was 0.73%. After 100 F–T cycles, the volume of detected pores increased to 153.89 mm^3^ and total porosity changed to 0.89%. This porosity is much lower than the total porosity calculated by standard methods. This indicates that most of the pore volume consists of pores smaller than 100 µm and corresponds to MIP results. Because of the used voxel size of 34 µm/voxel, we were able to scan only pores with an effective diameter bigger than 136 µm. On this basis, we can state that most porosity is mainly built up by pores smaller than 0.1 mm^3^ (Fig. 7).

Table 2 shows the overall increase in five porosity categories according to their ED pores > 3 mm; > 2 mm; > 1 mm; > 0.5 mm and > 0.1 mm.

**Table 2:** Porosity by µCT before and after 100 F–T cycles.

Effective diameter ED (mm)	0.2–0.5	0.5–1.0	1.0–2.0	2.0–3.0	> 3.0
Porosity before F–T (%)	0.7063	0.6643	0.6067	0.5626	0.5612
Porosity after F–T (%)	0.8685	0.8295	0.7796	0.7447	0.7467
Δ (%)	22.96	24.86	28.49	32.36	33.05

**Figure 8:**
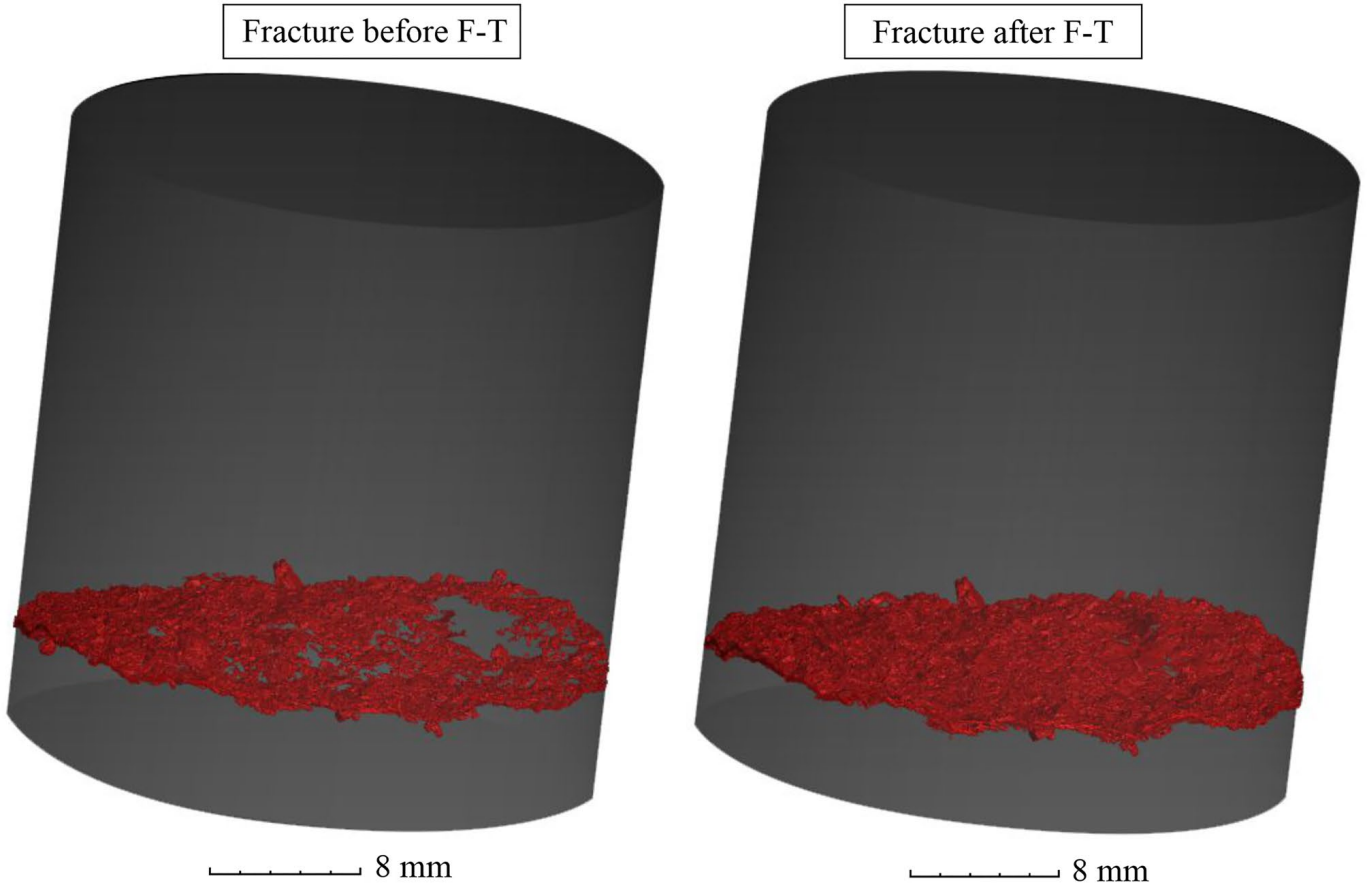
Visualization showing detail of transversal fracture and its volume increase after 100 F–T cycles by 31.44 pp.

Microcrack opening is one of the most important processes of rock disintegration, especially in andesitic rocks, since they normally exhibit good fabric cohesion. One single fracture running transversally to the sample with a total volume of 97.55 mm^3^ was detected and visualized. After 100 F–T cycles, fracture volume increased to 128.22 mm^3^ (Fig. 8).

The largest dimensional changes of the porous system were bound to and near the position of the fracture. This information provides complementary information about microcrack opening in this sample. The fracture grew perpendicular to the bedding of the andesite and the pore structure changed.

## Discussion

Changes in pore space properties induced by ice crystallization, which has been reported by various researchers, is a process that significantly affects the durability of rock material. According to Ruedrich et al.^42^ and Ruedrich and Siegesmund^14^, the mineralogical composition and fabric components control the stress that is developed in the pore space of the material. On the other hand, rock pore structure parameters, both geometric (porosity, pore size distribution, and shape of the pores) and topological (pore space interconnection), control the amount and distribution of moisture in the rock, and hence the possibility of ice crystallization. In this study, we focused on the behaviour of andesitic rock with a pre-existing crack under the cyclic 100 F–T cycles. Based on the changes in petrophysical properties, through the monitoring of strain behaviour, as well as temperature development and its comparison with non-destructive visualization using industrial µCT, we can outline a conclusion that may help clarify the degeneration of rock with a pre-existing fracture during frost weathering. The tested sample of andesite from the Babina Quarry is a rock which exhibits good fabric cohesion. Its porosity is extremely low, and its rock pore structure system is specific and complicated. According to MIP results, its pore size distribution pattern is bimodal, with a large number of macro and mesopores, which are interconnected by micropores. Stresses which developed during the ice crystallization led to dimensional changes in its pore structure. According to Sun and Sherer^43^, at lower temperatures, ice crystallization takes place in smaller pores, because the probability of having a good nucleation surface decreases with pore volume. In our study, a relatively intense expansion started at about − 0.5 °C in Zone II. This indicated the start of water crystallization within larger pores, which is more favorable according to equilibrium thermodynamics. Specimen expansion is probably induced by crystallization and hydraulic pressures inside the pore system. The growing crystals also draw unfrozen water towards them^4^, and this process is called cryosuction. The crystallization pressures are larger in smaller pores; however, for ice to penetrate smaller pores, the temperature needs to be lower. At − 0.5 °C, water in micropores remains liquid in metastable-supercooled condition and supports crystal growth in larger pores. As water crystallizes first in the larger pores of the system in a fast manner, the 9% volume expansion causes part of the water in these larger pores to be forced out into the smaller neighboring pores. Hence, if this expelled water cannot find a pore large enough, or pore interconnection is too low to relieve this pressure, hydraulic pressures then build up, and along with crystallization pressures, can exceed the cohesive strength of the material and cause cracks to extend. On the other hand, ice crystallization induced processes cause negative pore pressure in micropores, which means that the material shrinks instead of expanding in Zone III. Freezing of crack water to ice resulted in crack opening and subsequent fracture volume increase of 31 pp. This process decreased rock cohesive strength progressively and led to a residual strain of 8 × 10^–5^ after 100 F–T. Residual strain of 8 × 10^–5^ is not large enough to be macroscopically observable on the specimen, but it is sufficient for a relatively high increase in porosity, pore interconnection, and changes in the pore size distribution pattern of the rock. Such progressive deterioration of the properties of the rock material is called subcritical crack growth. In a recent review of subcritical crack growth mechanics, Eppes and Keanini^2^ argue that climate-dependent subcritical microcrack expansion is a potentially responsible process for the growth of most cracks in surface and near-surface rocks. Subcritical crack growth is affected by many known, as well as anticipated chemical and physical processes associated with climate influence. Therefore, climate change will alter the frequency and magnitude of weathering processes, and thus the current understanding of frost weathering is required to anticipate future occurrence of processes which result in microcrack propagation and the resulting deterioration of building materials, natural stones, or freeze–thaw triggered rockfalls^44^.

## Conclusions

Imaging applied to multiple µCT images before and after 100 F–T cycles, which were performed on a vacuum-saturated sample, allows for non-destructive visualizations of the rock pore structure evolution in the andesite. In combination with the study of volume modification by changes in petrophysical properties, as well as the recorded strain path behaviour with temperature development by the specially constructed thermodilatometer VLAP 04 with two HIRT-LVDT sensors, we can make conclusions regarding the effective mechanism of rock material deterioration in additional to commonly used standard methods. Based on our results it can be stated:

- The tested andesite from Babiná is a rock with extremely low porosity and a specific pore size distribution pattern with a large number of small capillary pores and micropores, as well as a large amount of macropores. This corresponds to the results from the indicative rock pore structure method. Based on those results, the rock pore structure of Babina andesite predominantly contains hardly-accessible macropores, which are interconnected by micropores and mesopores. A part of this specimen’s matrix contains a large amount of blind and isolated pores. Pore interconnection determined by the imbibition curve slope C(I) is also extremely low, but with a significant increase after F–T cycling.
- non-destructive visualization by µCT showed only a slight increase in macroporosity of the sample after 100 F–T cycles. On the other hand, significant fracture opening corresponds to a 31 pp. increase of fracture volume. The largest dimensional changes of porous structures are also bound to the locations near the fracture.
- the physical breakdown of rock necessarily stems from the propagation of fractures. Freeze–thaw induced cracking of a brittle-elastic solid like pyroxene-andesite is caused by ice crystallization and hydraulic pressure build-up which led to rock fatigue failure. Subcritical cracking of the tested andesite results in total residual strain of 8 × 10^–5^ recorded after 100 F–T cycles.
